# Tunable in-plane conductance anisotropy in 2D semiconductive AgCrP_2_S_6_ by ion-electron co-modulations

**DOI:** 10.1126/sciadv.adr3105

**Published:** 2025-01-08

**Authors:** Yujie Sun, Rongjie Zhang, Junyang Tan, Shengfeng Zeng, Shengnan Li, Qiang Wei, Zhi-Yuan Zhang, Shilong Zhao, Xiaolong Zou, Bilu Liu, Hui-Ming Cheng

**Affiliations:** ^1^ Shenzhen Geim Graphene Center, Shenzhen Key Laboratory of Advanced Layered Materials for Value-added Applications, Tsinghua-Berkeley Shenzhen Institute and Institute of Materials Research, Tsinghua Shenzhen International Graduate School, Tsinghua University, Shenzhen 518055, PR China.; ^2^ School of Electronic Information Engineering, Foshan University, Foshan 528000, PR China.; ^3^ Institute of Technology for Carbon Neutrality, Shenzhen Institute of Advanced Technology, Chinese Academy of Sciences, Shenzhen 518055, PR China.; ^4^ Shenyang National Laboratory for Materials Science, Institute of Metal Research, Chinese Academy of Sciences, Shenyang 110016, PR China.

## Abstract

In-plane anisotropic two-dimensional (2D) semiconductors have gained much interest due to their anisotropic properties, which opens avenues in designing functional electronics. Currently reported in-plane anisotropic semiconductors mainly rely on crystal lattice anisotropy. Herein, AgCrP_2_S_6_ (ACPS) is introduced as a promising member to the anisotropic 2D semiconductors, in which, both crystal structure and ion-electron co-modulations are used to achieve tunable in-plane conductance anisotropy. Scanning tunneling electron microscopy and polarized Raman spectroscopy show the structural anisotropy of ACPS. Electrical transport measurements show that its tunable in-plane conductance anisotropy is related to the ion-electron co-modulations, where Ag ion migration is anisotropic along *a* axis and *b* axis. Electrical transport measurements show the semiconducting properties of ACPS, as also supported by photoluminescence results. Moreover, the transfer curves of ACPS showcase large *V*_g_-related hysteresis, which is directionally controlled by anisotropic Ag ion migration. This work offers a possibility of using anisotropic charge transport in functional electronics by ion-electron co-modulations.

## Teaser

The electrical transport of 2D anisotropic materials can be tunable by ion-electron co-modulations.

## INTRODUCTION

Two-dimensional (2D) materials (*1*, *2*) are characterized by anisotropic structures and properties between in-plane and out-of-plane directions (*3*). Anisotropy within the in-plane is also found in some 2D materials, giving an additional degree of freedom to tune their electrical, optical, and thermal behaviors, which is not possible for in-plane isotropic 2D materials. For instance, black phosphorous shows in-plane conductance anisotropy with a carrier mobility ratio of 1.5 between directions of armchair and zigzag (*4*). In addition, it shows in-plane anisotropic thermal conductivity along armchair and zigzag directions (*5*). This in-plane anisotropy in electrical/thermal property can be used in directional transport of charge and energy (*6*). Thus, in-plane anisotropy opens an avenue to design functional electronic devices (*7*–*9*), such as anisotropic transistors (*10*, *11*), anisotropic artificial synapses (*12*), and anisotropic photodetectors (*13*–*15*). More and more in-plane anisotropic 2D materials are explored recently, such as black arsenic (*16*), ReS_2_ (*17*), Sb_2_Se_3_ (*18*), GeP (*19*), and TaIrTe_4_ (*20*). So far, most reported in-plane conductance anisotropy ratio is fixed with the orientation of crystal lattice, which is limited in achieving tunable in-plane conductance anisotropy. Introducing a new degree of freedom to tune in-plane conductance anisotropy may further promote the development of anisotropic electronics.

To achieve tunable in-plane conductance anisotropy of a 2D material, intrinsic ion modulation within the material is a potential choice as it can couple with electrons (*21*) to tune device conductance (*22*–*25*). For instance, Zhang *et al.* (*24*) used the migration of intrinsic K ion inside mica to realize the non-Markov chain. Chen *et al.* (*25*) used the migration of intrinsic Cu ion inside CuInP_2_S_6_ to generate continuous variable resistance states, and achieved synaptic plasticity. As intrinsic ion is the inherent component of the crystal structure, in-plane conductance anisotropy is tunable by directionally arranging intrinsic ion inside the material. In this regard, Wang *et al.* (*26*) recently showed that the arrangement of intrinsic Cu ion in insulated CuCrP_2_S_6_ is in-plane anisotropic, achieving directional conductance change. Furthermore, in-plane anisotropic ion migration may couple with gate tunability in 2D semiconductors, which may lead to ion-electron co-modulations in three-terminal–based charge transport. To our best knowledge, it has not been reported yet.

Here, we report such a device that achieves conductance tuning via coupling of anisotropic ion migration and electrical gating in a 2D semiconductive AgCrP_2_S_6_ (ACPS). Scanning tunneling electron microscopy (STEM) and polarized Raman spectroscopy results confirm the in-plane anisotropy of ACPS. The photoluminescence and electrical transport characteristics of ACPS indicate that it is a semiconductor. Ag ions in ACPS are proved to be moveable, generating anisotropic regulation of conductance from two-terminal current-voltage relation (*I-V*) measurements. The ACPS device generates a *V*_g_-related hysteresis in transfer curves, as Ag ions may couple with transporting electrons, leading to ion-electron co-modulations in a three-terminal mode. Moreover, anisotropic migration of Ag ions is used to directionally change the *V*_g_-related hysteresis windows in transfer characteristics with increased *V*_ds_. Therefore, the electrical behavior of ACPS is tuned by combining anisotropic migration of intrinsic Ag ions and semiconducting properties along *a* axis and *b* axis of ACPS. Our work provides an insight in designing devices with functionalities by coupling with in-plane ionic anisotropy.

## RESULTS

### Structure characterization of 2D semiconductive ACPS

**Fig. 1.**
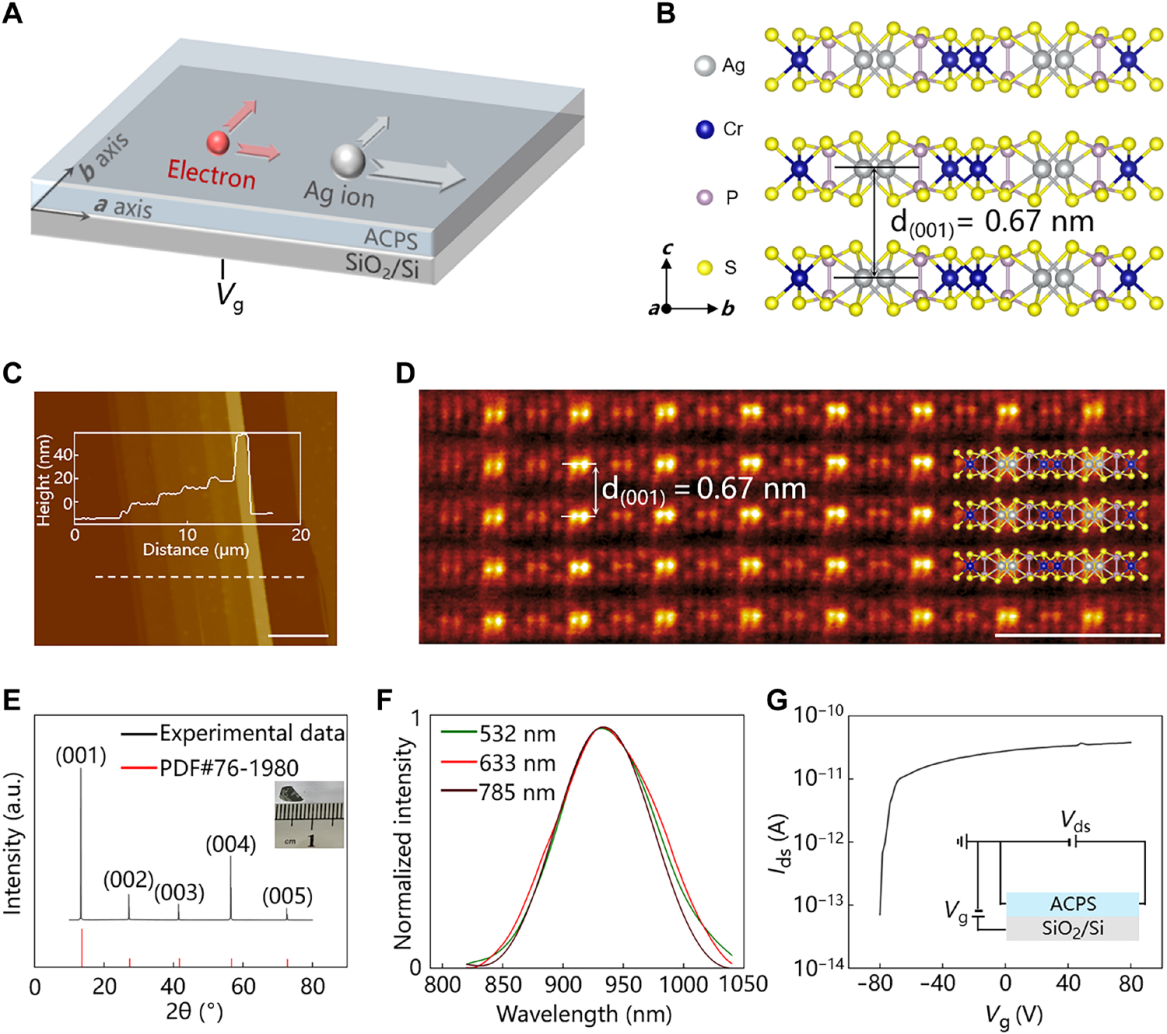
Structure characteristics of 2D semiconductive ACPS. (**A**) Schematic of tunable in-plane anisotropy in the ionic semiconductor ACPS. (**B**) Side-view crystal structure of ACPS. (**C**) AFM image of an ACPS flake. Scale bar, 5 μm. Inset is the height profile along the white dotted line. (**D**) Cross-sectional HAADF-STEM image of ACPS. Scale bar, 2 nm. (**E**) X-ray diffraction pattern of an ACPS flake. Inset is the photo of the grown ACPS crystal. (**F**) Photoluminescence spectra of an ACPS flake. (**G**) Transfer curve of an ACPS flake. *V*_ds_ = 1 V.

Figure 1A shows the schematic of ion-electron co-modulations design based on in-plane ionic anisotropy in ACPS. Figure 1B depicts the side-view atomic arrangement of ACPS. It shows a monoclinic crystal with a layered structure. The lattice parameters are *a* = 5.9 Å, *b* = 10.6 Å, *c* = 6.7 Å, α = 90°, β = 106°, and γ = 90°. Ag atoms are inside the sulfur-based framework, and the adjacent layers interact via the van der Waals force. The ACPS crystal was grown by chemical vapor transport, where Ag, Cr, P, and S powders were used as precursors (see Materials and Methods for details). The Raman spectrum of ACPS shows peaks at 201.4, 268.2, 513.8, and 656.8 cm^−1^, assigned to the $A_{g}$ modes according to the first-principles calculations (figs. S1 and S2 and table S1) (*27*). Atomic force microscopy (AFM) image of the exfoliated ACPS flake (fig. S3) is shown in Fig. 1C, and the ACPS flake with different thicknesses of 6, 13, 20, 25, 32, and 72 nm was obtained. The cross-sectional high-angle annular dark-field STEM (HADDF-STEM) image (Fig. 1D) along [100] axis clearly shows the layered structure of ACPS and, in particular, the interplanar spacing of (001) crystal plane is 0.67 nm, which is consistent with that in Fig. 1B. The x-ray diffraction pattern (Fig. 1E) of ACPS shows five distinct peaks at 2θ = 13.3°, 27.3°, 41.6°, 56.5°, and 72.6°, which belong to (001), (002), (003), (004), and (005) crystal planes, respectively. Figure 1F shows the photoluminescence spectra of ACPS, indicating that ACPS has a photoluminescence peak at about 940 nm. Figure 1G showcases that the electrical transport of ACPS is gate tunable, indicating the semiconductive behaviors of ACPS. In brief, the results in the above characterizations show the layered structure of semiconductive ACPS.

### In-plane structural and optical anisotropy of ACPS

**Fig. 2.**
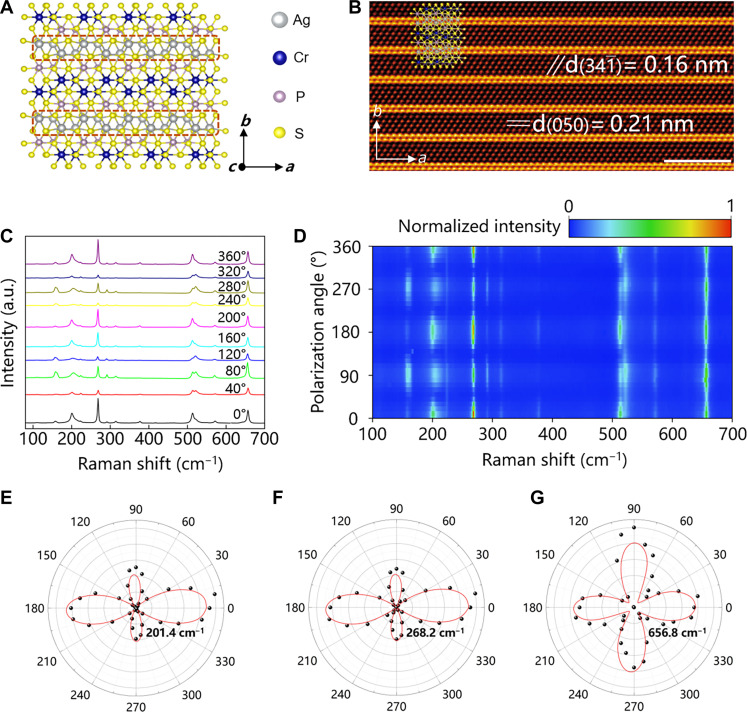
In-plane structural and optical anisotropy of ACPS. (**A**) Top-view crystal structure of ACPS. (**B**) HAADF-STEM image of ACPS with indications of d(050) and d(34$\bar{1}$). Inset is the overlapped crystal structure shown in (A). Scale bar, 2 nm. (**C**) Polarized Raman spectra of an ACPS flake. (**D**) Intensity map of 360° polarized Raman spectra. (**E** to **G**) Polar plots of Raman intensities of 201.4, 268.2, and 656.8 cm^−1^. The black dots represent the experimental results in polarized Raman spectroscopy, and the red solid lines correspond to the ideal curves.

We investigated the in-plane anisotropy of ACPS. Figure 2A shows the anisotropic crystal structure of ACPS in *ab* plane. The arrangement of Ag is continuous along *a* axis, while it is intermittent along *b* axis. This arrangement difference in two directions makes Ag atoms arrange like 1D ribbons in *ab* plane. The ACPS flake (fig. S4) was used in STEM characterization. The energy-dispersive x-ray spectroscopy (EDS) mapping of the ACPS flake proves the uniform element distribution of Ag, Cr, P, and S elements (figs. S5 and S6). The fast Fourier transform pattern of the ACPS flake is consistent with the simulated result (fig. S7). The presence of only one set of diffraction spots indicates its single-crystal nature. The HADDF-STEM image (Fig. 2B) of the ACPS flake along [103] axis shows the in-plane atomic arrangement of ACPS. The interplanar spacings of (34$\bar{1}$) and (050) crystal planes are 0.16 and 0.21 nm, respectively. The Ag atoms form 1D chains, which are separated by [CrPS] units, extending along the axial direction of the ACPS ribbon. We used the polarized Raman spectroscopy to characterize the optical anisotropy of an ACPS flake. The polarization angle of the incident light is indicated in fig. S8. The polarized Raman spectra of ACPS are shown in Fig. 2 (C and D). Figure 2 (E to G) shows the polar plots of Raman intensities of 201.4, 268.2, and 656.8 cm^−1^ in ACPS during the polarization rotation. On the basis of characterizations of STEM and polarized Raman spectroscopy, the in-plane anisotropy of ACPS is confirmed.

### In-plane anisotropic ion migration in ACPS

**Fig. 3.**
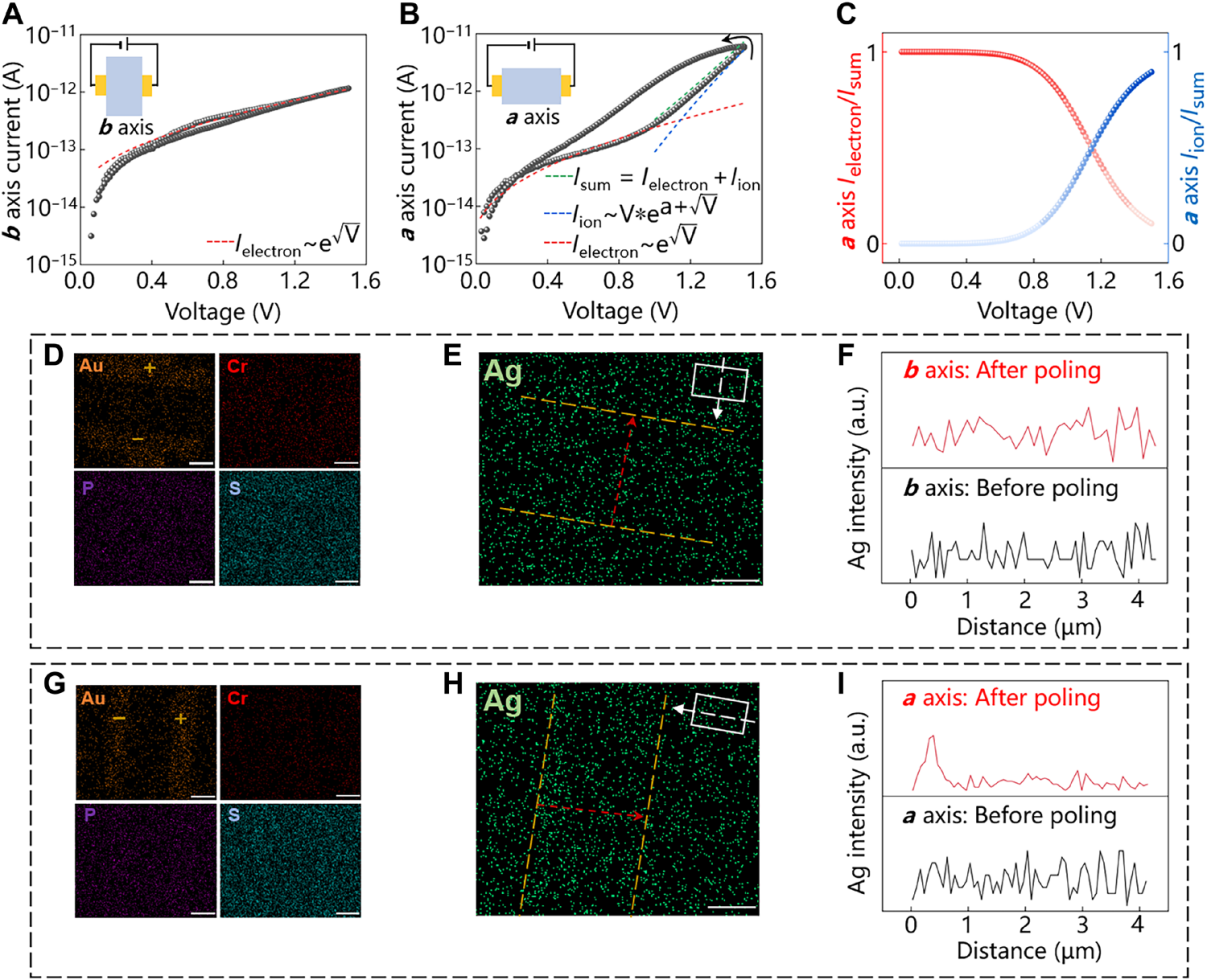
In-plane anisotropic ion migration in ACPS. (**A**) *I-V* curve along the *b* axis of the ACPS flake, without *V*_g_. Red dotted line is the fitting curve. The inset is the schematic of *I-V* measurements along the *b* axis. (**B**) *I-V* curve along the *a* axis of the ACPS flake, without *V*_g_. Red, green, and blue lines correspond to fitting curves. The inset is the schematic of *I-V* measurement along the *a* axis. (**C**) Comparison of current contribution between electron transport and ion migration along *a* axis of the ACPS flake. (**D**) EDS mappings of Au, Cr, P, and S along the *b* axis of the ACPS flake after electrical poling (10 V, 60 min). Scale bar, 2 μm. (**E**) EDS mapping of Ag along the *b* axis of the ACPS flake after electrical poling (10 V, 60 min). Scale bar, 2 μm. (**F**) EDS line scanning result of Ag, along with the red dotted arrow in (E). (**G**) EDS mappings of Au, Cr, P, and S along the *a* axis of the ACPS flake after electrical poling (10 V, 60 min). Scale bar, 2 μm. (**H**) EDS mapping of Ag along *a* axis of the ACPS flake after electrical poling (10 V, 60 min). Scale bar, 2 μm. Insets in (E) and (H) illustrate that the electric field is parallel to the *b* axis and *a* axis of the ACPS flake, respectively. (**I**) EDS line scanning of Ag, along with the red dotted arrow in (H).

Then, we studied the in-plane anisotropic Ag ion migration in ACPS. We fabricated two devices in an ACPS flake, along the *a* axis and *b* axis, respectively. Figure 3A shows the *I-V* curve of the device along *b* axis, without *V*_g_. When voltage is swept from 0 to 1.5 V, the current gradually increases. The bias-related hysteresis is small when voltage is swept back from 1.5 to 0 V. In contrast, the device along *a* axis shows two steps of current change under the same *I-V* measurement (Fig. 3B). The first current change is in the range from 0 to 1 V, which is similar to the current change of the device along *b* axis. However, the device along *a* axis generates a larger current increase when voltage exceeds 1 V compared with the *b* axis. An anticlockwise bias-related hysteresis is formed during the back scanning from 1.5 to 0 V, which is larger than that along *b* axis. As anticlockwise bias-related hysteresis is a typical characteristic in the setting process of ion transported device (*28*–*30*), this comparison indicates that the appearance of Ag ion migration along *a* axis is easier than that along *b* axis due to the anisotropic structure. To further analyze the contribution of electron transport and ion migration, Shockley emission model (*31*) and Poole-Frenkel model (*32*) are introduced. Shockley emission model is $I=AT^{2}e^{\frac{-\varphi }{\mathrm{kT}}}e^{\frac{\sqrt{V}}{\mathrm{kT}}\sqrt{\frac{q^{3}}{\mathrm{Ka}}}}$, used to describe the conduction based on moving electrons under an interfacial energy barrier. Poole-Frenkel model is $I\propto Ve^{\left(\sqrt{\frac{q^{3}V}{4\pi \varepsilon_{0}\varepsilon_{r}\mathrm{dkT}}}-\frac{q\varphi }{\mathrm{kT}}\right)}$, used to describe the conduction based on the assistance of ionized charges inside material. The *I-V* curve of the device along *b* axis is well fitted with the Shockley emission model, indicating the domination of electron transport. The *I-V* curve of the device along *a* axis in the range of 0 to 1 V is well fitted with the Shockley model, indicating that the current is mainly contributed from electron transport. The combination of the Shockley model and Poole-Frenkel model fits well in the range of 1 to 1.5 V. The current value is the sum of *I*_electron_ (Shockley model fitted) and *I*_ion_ (Poole-Frenkel model fitted), indicating the mixed conduction based on electron transport and ion migration. The current contributions of electron transport and ion migration along *a* axis of ACPS are compared in Fig. 3C. To further confirm the Ag migration processes, the elemental EDS mapping was characterized after electrical poling. All the elements are uniformly distributed in the channel of the device along *b* axis after poling (Fig. 3, D and E), and the EDS line scanning (Fig. 3F) shows no obvious Ag aggregation. The Cr, P, and S distributions in the channel of the device along *a* axis are uniform after poling (Fig. 3G). However, the distribution of Ag is changed as Ag ions aggregate on the left electrode under the stimulation of electrical field (Fig. 3H), which is consistent with the EDS line scanning (Fig. 3I). The EDS mappings of Ag, Cr, P, and S along *a* axis over time further indicate that only the distribution of Ag ions is gradually changed, which also prove the capability of Ag ion migration (figs. S9 and S10). Hence, directional tuning of conductance in ACPS is achieved on the basis of the anisotropic arrangement of Ag ions.

### Ion-electron co-modulations in anisotropic semiconductive ACPS

**Fig. 4.**
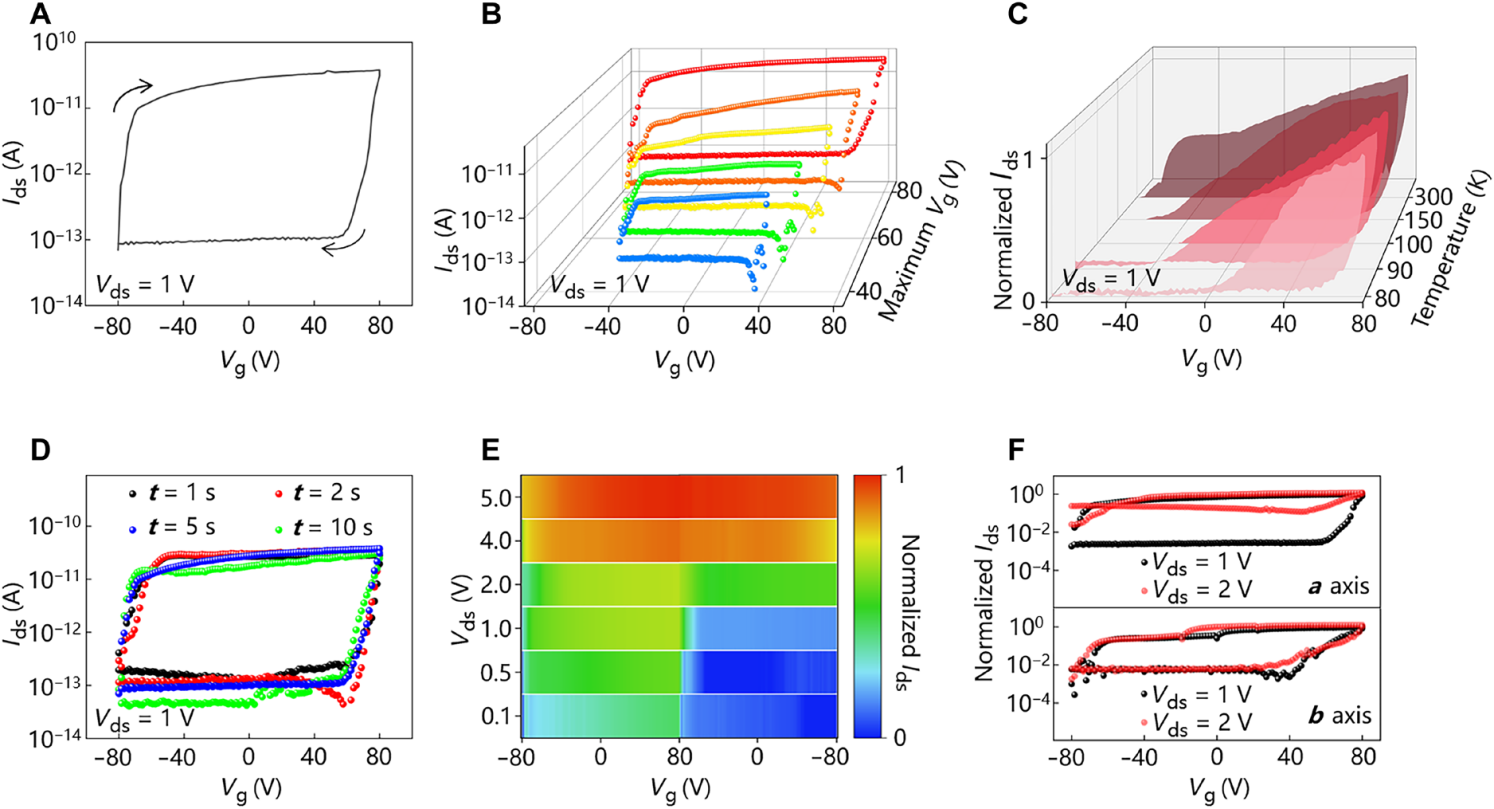
Ion-electron co-modulations in anisotropic semiconductive ACPS. (**A**) Transfer curve with a *V*_g_-related hysteresis of the ACPS device along *a* axis. (**B**) Transfer curves of the ACPS device along *a* axis with varying maximum *V*_g_. (**C**) Normalized transfer curves of the ACPS device along *a* axis at different temperatures of 300, 150, 100, 90, and 80 K. (**D**) Transfer curves of the ACPS device along *a* axis with different applied pulse time (*t*) of each *V*_g_ point. (**E**) Heatmap of transfer curves along the *a* axis of the ACPS device with different *V*_ds_, which is converted from fig. S13. (**F**) Normalized transfer curves along the *a* axis and *b* axis of the ACPS flake with different applied *V*_ds_.

Last, we studied the anisotropic Ag ion migration of ACPS in electrostatic gate tuning by a three-terminal configuration. Figure 4A shows the transfer curve of an ACPS device along *a* axis, where a large *V*_g_-related hysteresis is clearly observed. The *V*_g_-related hysteresis also appears in different sweep ranges of *V*_g_ (Fig. 4B and fig. S11) and along *b* axis (fig. S12). We further analyzed the *V*_g_-related hysteresis behavior at different temperatures, as the temperature-dependent *V*_g_-related hysteresis window change is helpful to analyze the mechanism of *V*_g_-related hysteresis generation (*33*–*35*). Figure 4C shows that the window of *V*_g_-related hysteresis is strongly affected by temperature change. It indicates that the generation of *V*_g_-related hysteresis may relate to electron trapping by charged ion activation. Figure 4D shows the transfer curve with *V*_ds_ = 1 V, with different sampling time (*t*) of each *V*_g_ point. For instance, the total measuring time of the transfer curve is around 202 s in the case of *t* = 1 s, while it is around 2020 s in the case of *t* = 10 s, which is sufficient for ion migration (*30*, *36*). It is observed that the *V*_g_-related hysteresis is independent with *t*, indicating that ion migration does not contribute to the formation of *V*_g_-related hysteresis. Moreover, further increasing the *V*_ds_ can induce different transfer characteristics in ACPS due to the migration of Ag ions. Figure 4E shows the heatmap of transfer curves by changing V_ds_. The *V*_g_-related hysteresis is large as the color change sharply near the *V*_g_ of 80 V when *V*_ds_ is lower than 1 V. Ag ions begin to be moveable when *V*_ds_ is larger than 1 V, diminishing the *V*_g_-related hysteresis, as Ag ion migration is used to tune the on/off ratio of transfer curve. Besides, the anisotropic migration of Ag ions in the ACPS offers the possibility to tune *V*_g_-related hysteresis, which exhibits unique functions compared with conventional architectures. The directional tuning of *V*_g_-related hysteresis is achieved by Ag ion migration and structural anisotropy (Fig. 4F). The above electrical measurements show that the electrical behavior of the ACPS device is tunable on the basis of coupling of in-plane anisotropic ion migration and semiconducting properties, which is rarely reported in materials with similar structures. Therefore, it combines in-plane ionic anisotropy and semiconductivity to achieve tunable in-plane conductance anisotropy in a three-terminal mode.

## DISCUSSION

We demonstrated anisotropic ion modulation and gate-tunable ion-electron coupling behavior in a 2D material ACPS, which is a semiconductor with in-plane ionic anisotropy. STEM and polarized Raman spectroscopy confirm the in-plane anisotropy of ACPS. Because of the ion-electron mixed conducting mechanism, the anisotropic conductance change of two-terminal ACPS device is achieved by anisotropic Ag ion migration. EDS mapping shows the discrepancy of Ag ions migrating along the *a* axis and *b* axis of ACPS. As an ionic semiconductor, the ACPS device exhibits a large *V*_g_-related hysteresis. Moreover, *V*_g_-related hysteresis is directionally tuned on the basis of the anisotropic Ag ion migration in ACPS. The results show the possibilities of ion-electron co-modulation by coupling of in-plane ionic anisotropy and semiconducting properties, suggesting a new degree of freedom in anisotropic electronics.

## MATERIALS AND METHODS

### Materials preparation and device fabrication

We used chemical vapor transport to grow the ACPS crystal. In brief, Ag, Cr, P, and S powders were sealed in a quartz tube (10^−4^ mbar) with a mole ratio of 1:1:2:6. The quartz tube was firstly heated up to 400°C in 10 hours and held for 24 hours. Then, it was heated up to 750°C in 14 hours and held for 1 week. Last, the ACPS crystal was obtained after the cooling of quartz tube. ACPS crystal was exfoliated by a scotch tape and transferred onto a SiO_2_/Si substrate by polydimethylsiloxane. To deposit electrodes, a photoresist [poly(methyl methacrylate) A6 950K] was spin-coated (2000 rpm, 1 min) to uniformly cover the SiO_2_/Si substrate. After that, it was baked at 165°C for 1 min. Electron beam lithography processes was used to pattern the electrodes (Raith, German). Cr/Au (10 nm/20 nm) was deposited by electron-beam evaporation at a base pressure of 10^−4^ Pa (TSV-1500, Tianxingda Vacuum Coating Equipment Co. Ltd., China).

### Material characterizations and device measurements

Photoluminescence measurements were performed with 532-, 633-, and 785-nm lasers (Horiba LabRABHR Evolution, Japan). Raman spectroscopy and angle-resolved polarized Raman spectroscopy were performed with a 633-nm laser (Horiba LabRABHR Evolution, Japan). The morphology of ACPS was characterized by AFM (Oxford Cypher ES, USA). A double-Cs–corrected TEM (FEI Spectra 300, USA) was used for microscopy analysis. Scanning electron microscopy (SEM) characterization was processed at an electron voltage of 10 kV (Sigma 300, Zeiss, German). To perform SEM/EDS observation of elemental distributions in ACPS after electric poling, the exfoliated ACPS flake was first deposited with patterned electrodes by e-beam lithography and e-beam evaporation. The source meter (Keithley 2400), connected to the electrodes by wire bonding, supplied voltage for electrical poling. Electrical measurements were performed in a probe station (Lakeshore TTPX, USA) with the vacuum of 10^−5^ mbar. The electrical curves were collected by a semiconductor parameter analyzer (Keithley 4200, USA).

#### I-V curve fitting

Shockley emission model is $I=AT^{2}e^{\frac{-\varphi }{\mathrm{kT}}}e^{\frac{\sqrt{V}}{\mathrm{kT}}\sqrt{\frac{q^{3}}{\mathrm{Ka}}}}$, where *A* is the Richardson constant, *T* is the Kelvin temperature, φ is the barrier height, *k* is the Boltzmann constant, *q* is the charge of electron, *K* is relative dielectric constant, and *ɑ* is the thickness of material. The corresponding expression of fitting curve can be written as $I=b_{1}e^{c_{1}\sqrt{V}}$, where $b_{1}=AT^{2}e^{\frac{-\varphi }{\mathrm{kT}}}$ and $c_{1}=\frac{1}{\mathrm{kT}}\sqrt{\frac{q^{3}}{\mathrm{Ka}}}$. Poole-Frenkel model is $I\propto Ve^{\left(\sqrt{\frac{q^{3}V}{4\pi \varepsilon_{0}\varepsilon_{r}\mathrm{dkT}}}-\frac{q\varphi }{\mathrm{kT}}\right)}$, where *q* is the charge of electron, ε_0_ is the vacuum permittivity, ε*_r_* is the relative permittivity, *d* is the thickness of material, φ is the barrier height, *k* is the Boltzmann constant, and *T* is the Kelvin temperature. The corresponding expression of fitting curve can be written as $I\propto Ve^{\left(b_{2}\sqrt{V}+c_{2}\right)}$, where $b_{2}=\sqrt{\frac{q^{3}}{4\pi \varepsilon_{0}\varepsilon_{r}\mathrm{dkT}}}$ and $c_{2}=-\frac{q\varphi }{\mathrm{kT}}$.
