# Supplementary material for: Tunable in-plane conductance anisotropy in 2D semiconductive AgCrP2S6 by ion-electron co-modulations
Source: Sci Adv. 2025 Jan 8;11(2):eadr3105. doi: 10.1126/sciadv.adr3105 (PMC11708896; doi:10.1126/sciadv.adr3105)
Supplement: Supplementary file 1 — Figs. S1 to S13 Table S1 Supplementary Text [file sciadv.adr3105_sm.pdf]

Science Advances
AAAS

Supplementary Materials for

Tunable in-plane conductance anisotropy in 2D semiconductive $AgCrP_2S_6$ by ion-electron co-modulations

Yujie Sun *et al.*

Corresponding author: Bilu Liu, bilu.liu@sz.tsinghua.edu.cn; Hui-Ming Cheng, cheng@imr.ac.cn

Sci. Adv. **11**, eadr3105 (2025)
DOI: 10.1126/sciadv.adr3105

This PDF file includes:

Figs. S1 to S13
Table S1
Supplementary Text

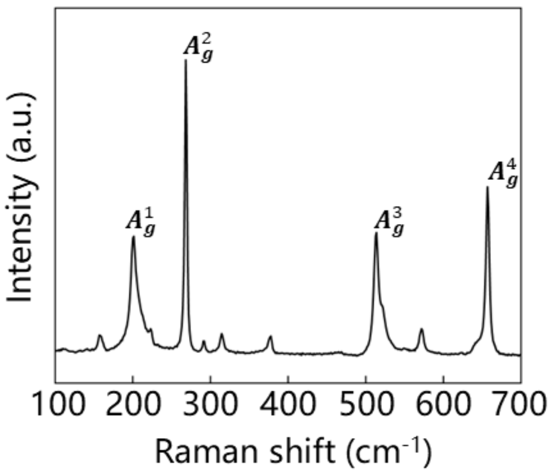

Figure S1. Raman spectrum of a $AgCrP_2S_6$ (ACPS) flake. The related vibration modes are labeled based on first principles calculations. ACPS belongs to C_{2h} point group symmetry, which exhibits four symmetry operations. The character table for C_{2h} includes four irreducible representations of A_g, A_u, B_g and B_u, with E, C_2, σ_h and i being identity, two-fold rotational symmetry, mirror symmetry and inversion symmetry operations, respectively (Table S1). The notation A is symmetric around its rotation center, while B is anti-symmetric around its rotation center. The notations g and u indicate representations are even and odd under inversion, respectively.

Table S1. Character table for group C_{2h}

C_{2h} (2/m)			E	C_2	σ_h	i
x^2, y^2, z^2, xy	R_z	A_g	1	1	1	1
	z	A_u	1	1	−1	−1
xz, yz	R_x, R_y	B_g	1	−1	−1	1
	x, y	B_u	1	−1	1	−1

Since there are 20 atoms in the unit cell, there are in total 60 representations, which can be decomposed into $\Gamma = 14A_g + 14A_u + 28B_g + 28B_u$. To estimate whether a representation is Raman active or not, the Raman scattering selection rule determined by symmetries of lattice vibrations is considered. For any symmetry operation R, there exists a transformation matrix denoted by:

$$\begin{pmatrix} R_{xx} & R_{xy} & R_{xz} \\ R_{yx} & R_{yy} & R_{yz} \\ R_{zx} & R_{zy} & R_{zz} \end{pmatrix} = \begin{pmatrix} cos\theta_R & -sin\theta_R & 0 \\ sin\theta_R & cos\theta_R & 0 \\ 0 & 0 & \pm 1 \end{pmatrix}$$

The selection rule shows that if an irreducible representation *i* is included in the basis of polarizability, the corresponding vibrational mode for *i* is Raman active. The criterion can be written in the form of:

$$\sum_R [2cos\theta_R(\pm 1 + 2cos\theta_R)]X^{(i)}(R) \neq 0$$

Where *X* is the character of symmetry operation R for the irreducible representation *i*. Transformation matrices for four different symmetry operations are:

$$E: \begin{pmatrix} 1 & 0 & 0 \\ 0 & 1 & 0 \\ 0 & 0 & 1 \end{pmatrix} C_2: \begin{pmatrix} -1 & 0 & 0 \\ 0 & -1 & 0 \\ 0 & 0 & 1 \end{pmatrix} I: \begin{pmatrix} -1 & 0 & 0 \\ 0 & -1 & 0 \\ 0 & 0 & -1 \end{pmatrix} \sigma_h: \begin{pmatrix} 1 & 0 & 0 \\ 0 & -1 & 0 \\ 0 & 0 & -1 \end{pmatrix}$$

Then, we can determine the Raman activity for four representations.

For A_g mode, $X^{A_g}(E) = 1, X^{A_g}(C_2) = 1,\ X^{A_g}(\sigma_h) = 1,\ X^{A_g}(i) = 1,$

$$\boldsymbol{A_g: 2(1+2) - 2(1-2) - 2(-1-2) + 2(-1+2) = 6 + 2 + 6 + 2 \neq 0}$$

For B_g mode, $X^{B_g}(E) = 1, X^{B_g}(C_2) = -1,\ X^{B_g}(\sigma_h) = -1,\ X^{B_g}(i) = 1,$

$$\boldsymbol{B_g: 2(1+2) + 2(1-2) - 2(-1-2) - 2(-1+2) = 6 - 2 + 6 - 2 \neq 0}$$

For A_u mode, $X^{A_u}(E) = 1, X^{A_u}(C_2) = 1,\ X^{A_u}(\sigma_h) = -1,\ X^{A_u}(i) = -1,$

$$\boldsymbol{A_u: 2(1+2) - 2(1-2) + 2(-1-2) - 2(-1+2) = 6 + 2 - 6 - 2 = 0}$$

For B_u mode, $X^{B_u}(E) = 1, X^{B_u}(C_2) = -1,\ X^{B_u}(\sigma_h) = 1,\ X^{B_u}(i) = -1,$

$$\boldsymbol{B_u: 2(1+2) + 2(1-2) + 2(-1-2) + 2(-1+2) = 6 - 2 - 6 + 2 = 0}$$

where A_g and B_g are Raman active while A_u and B_u are not.

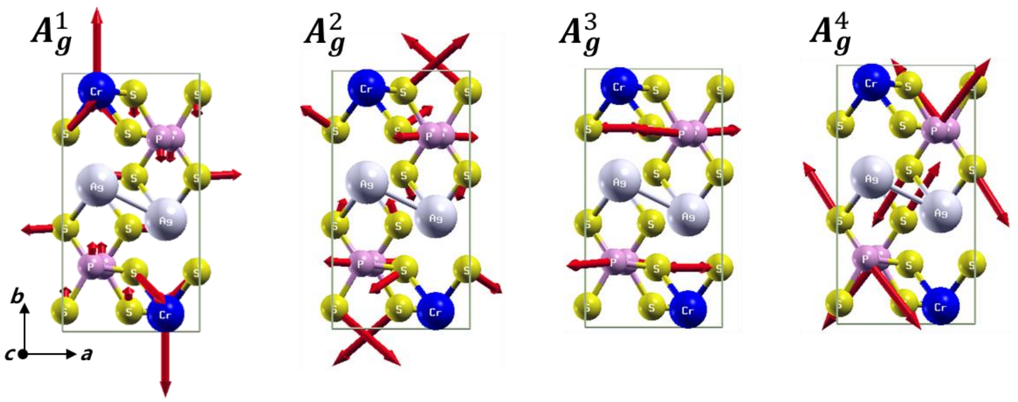

Figure S2. The schematics of vibration modes of A_g^1, A_g^2, A_g^3, and A_g^4 in ACPS.

The corresponding Raman tensor of A_g in C_{2h} point group symmetry can be written as:

$$\boldsymbol{R}(A_g) = \begin{pmatrix} \boldsymbol{a} & 0 & \boldsymbol{d} \\ 0 & \boldsymbol{b} & 0 \\ \boldsymbol{d} & 0 & \boldsymbol{c} \end{pmatrix}$$

Where $\boldsymbol{a}$, $\boldsymbol{b}$, $\boldsymbol{c}$, and $\boldsymbol{d}$ are matrix elements of the Raman tensor. Raman tensor elements of A_g can be denoted as $\boldsymbol{a} = |a|e^{i\varphi_a}$, $\boldsymbol{b} = |b|e^{i\varphi_b}$, $\boldsymbol{c} = |c|e^{i\varphi_c}$, and $\boldsymbol{d} = |d|e^{i\varphi_d}$ where φ_a, φ_b, φ_c and φ_d are the corresponding phases of the Raman tensor elements. The intensity of the Raman modes can be expressed as:

$$I \propto |\boldsymbol{e}_i \boldsymbol{R} \boldsymbol{e}_s|^2$$

Where $\boldsymbol{e}_i$ and $\boldsymbol{e}_s$ are the unit polarization vectors of incident and scattered lights, respectively, and $\boldsymbol{R}$ stands for the Raman tensor. The unit polarization vector $\boldsymbol{e}_i = (\cos\theta, \sin\theta, 0)$ and $\boldsymbol{e}_s = (\cos\theta, \sin\theta, 0)$, where θ is the angle between incident light polarization direction and x-axis, with crystallographic $\boldsymbol{a}$- and $\boldsymbol{b}$-axis defined as the x- and y-axis, respectively. The anisotropic Raman scattering intensities in parallel configuration can be written as:

$$I(A_g) = |a|^2 \left\{ \left(sin^2\theta + \left|\frac{b}{a}\right| cos\varphi_{ba} cos^2\theta \right)^2 + \left(\left|\frac{b}{a}\right| sin\varphi_{ba} cos^2\theta \right)^2 \right\}$$

Here, $\varphi_{ba} = \varphi_b - \varphi_a$ is the phase difference.

The main-axis direction of A_g depends on the values of $\left|\frac{b}{a}\right|$, and φ_{ba} is related to the strength of the secondary maximum on A_g.

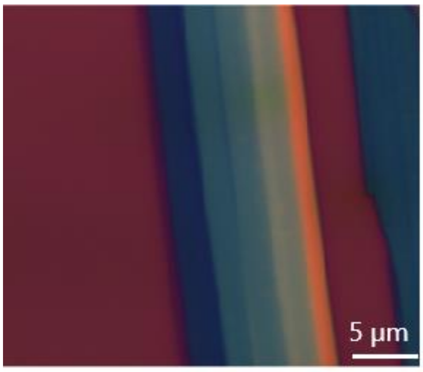

Figure S3. Optical image of an exfoliated ACPS flake.

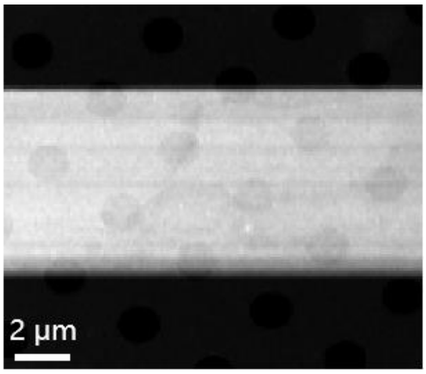

Figure S4. Low-magnification high-angle annular dark-field scanning tunneling electron microscopy image of the ACPS flake.

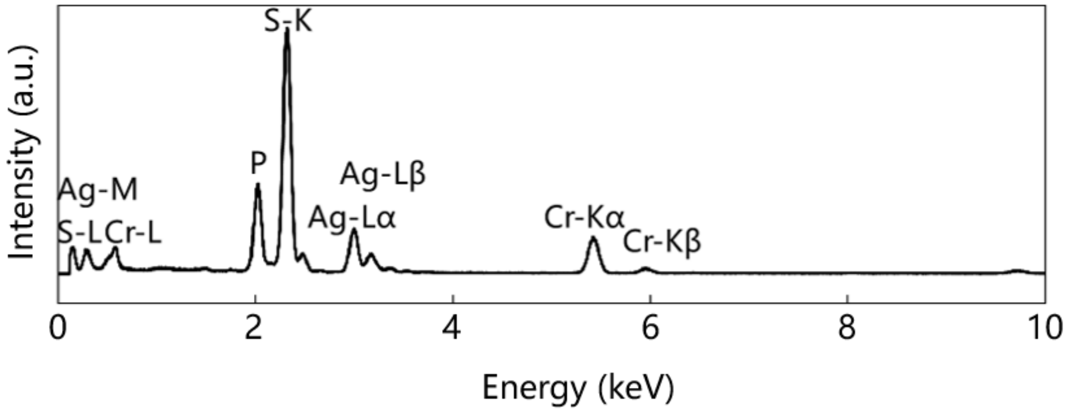

Figure S5. Energy dispersive spectrum of the ACPS flake, showing the existence of Ag, Cr, P, and S element.

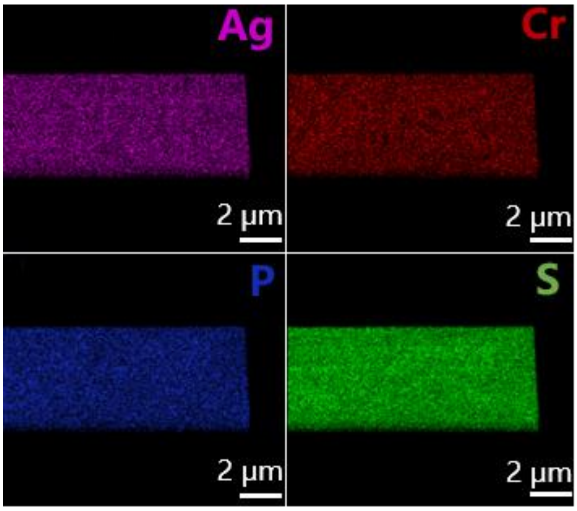

Figure S6. Energy-dispersive X-ray spectroscopy mapping of an ACPS flake.

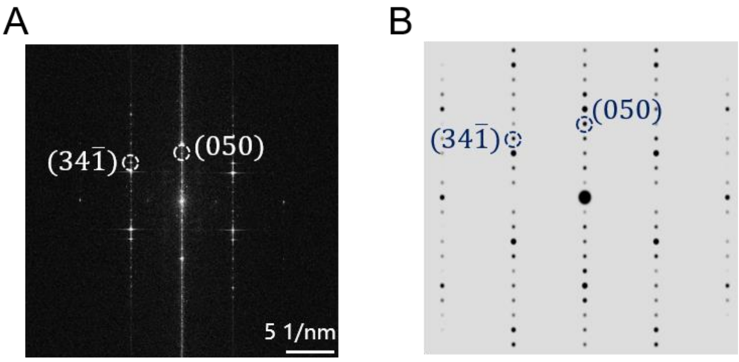

Figure S7. Fast Fourier transform pattern and simulated selected area electron diffraction pattern of an ACPS flake along [103] axis. (A) Fast Fourier transform pattern of an ACPS flake along [103] axis. (B) Simulated selected area electron diffraction pattern of an ACPS flake along [103] axis.

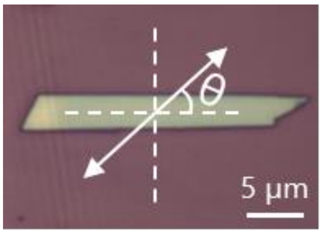

Figure S8. Optical image of an ACPS flake in the polarized Raman spectroscopy measurements.

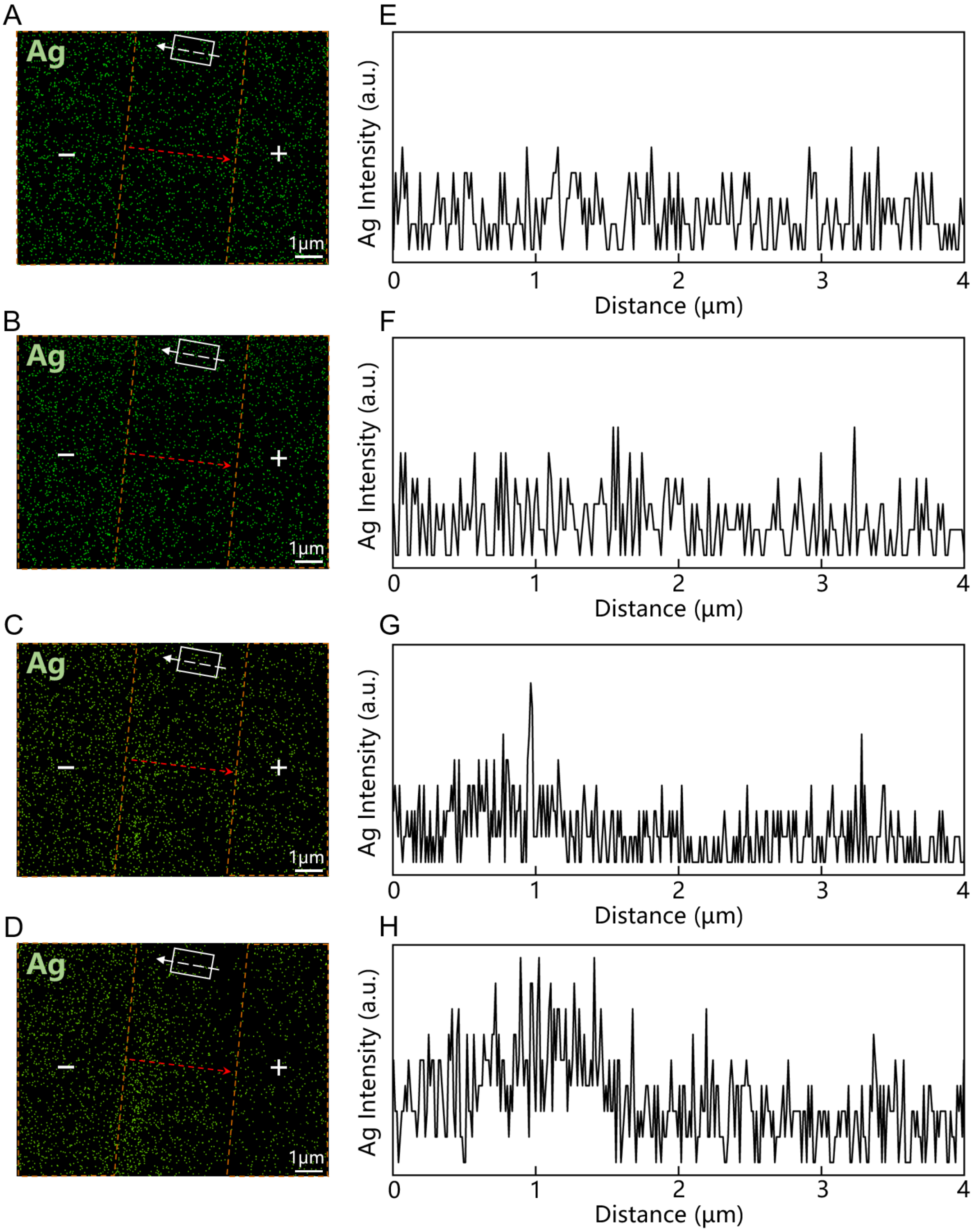

Figure S9. EDS mappings of Ag along the *a*-axis of ACPS device over time. (A-D) EDS mappings of Ag at initial state (A), and after 10 V poling for (B) 20 mins, (C) 40 mins, and (D) 60 mins, respectively. The insets in (A-D) indicate that the electric field direction is parallel to ***a***-axis. (E-H) EDS line scans of Ag along the red dotted arrow at initial state (E), and after 10 V poling for (F) 20 mins, (G) 40 mins, and (H) 60 mins.

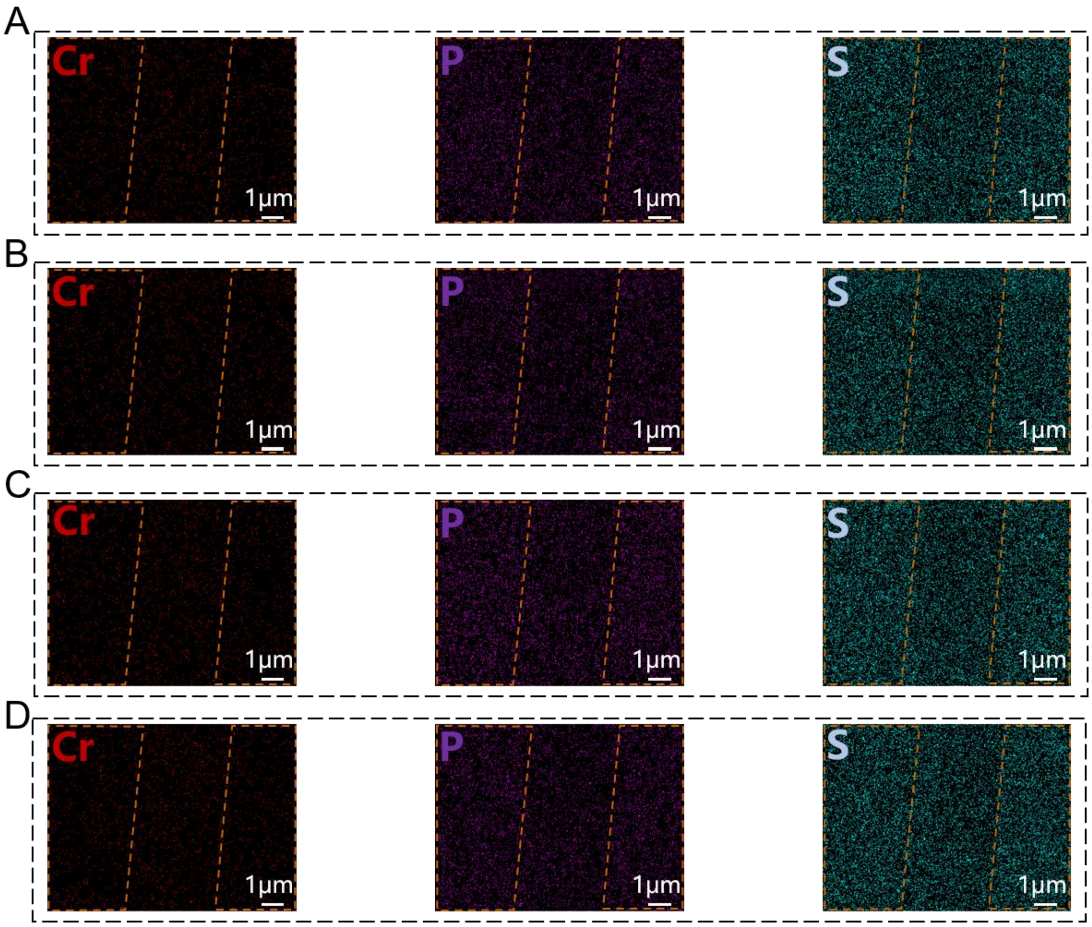

Figure S10. EDS mappings of Cr, P, and S along the *a*-axis of ACPS device over time. (A-D) EDS mappings of Cr, P, and S at initial state (A), and after 10 V poling for (B) 20 mins, (C) 40 mins, and (D) 60 mins, respectively.

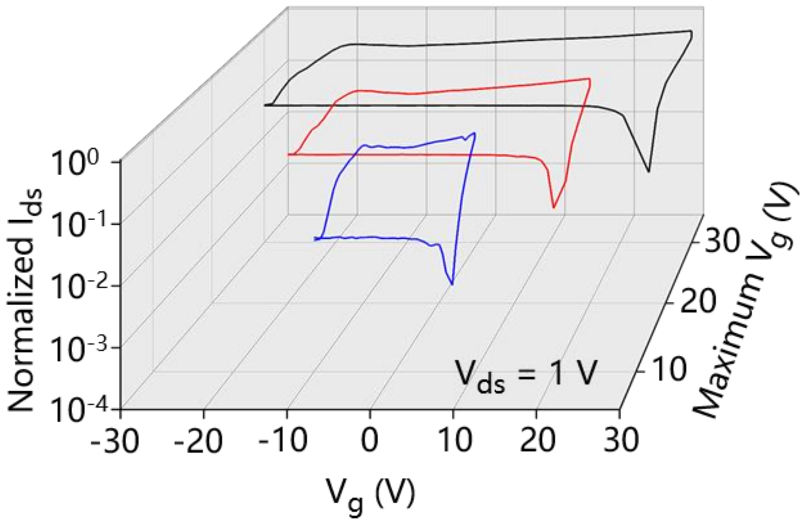

Figure S11. Normalized transfer curves of the ACPS device along *a*-axis of ACPS crystal.

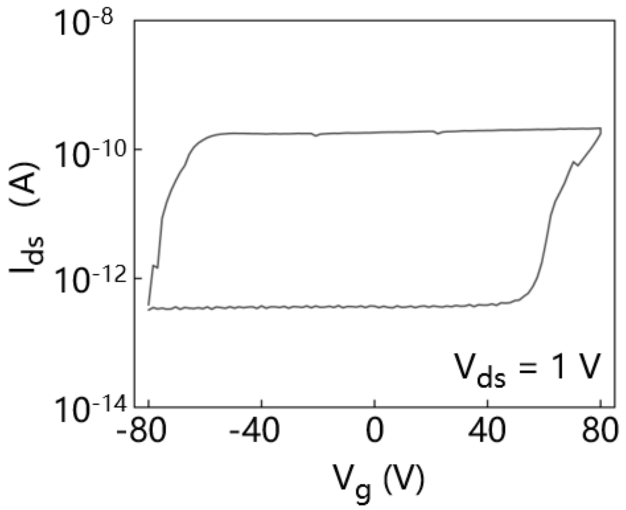

Figure S12. Transfer curve of the ACPS device along *b*-axis of ACPS crystal.

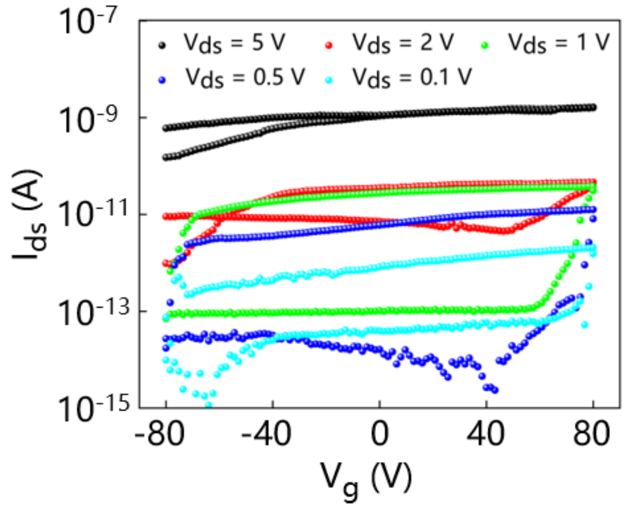

Figure S13. Transfer curves of the ACPS device along *a*-axis of ACPS flake with different V_{ds}.
